# RET@Thirty: the story behind identification of RET mutation as the cause of MEN2 and what it meant to clinical practice

**DOI:** 10.1530/ERC-24-0226

**Published:** 2025-02-24

**Authors:** Bruce Ponder, Tom R Kurzawinski

**Affiliations:** ^1^ Emeritus Professor of Oncology and Emeritus Director, Cancer Research UK Cambridge Institute, Cambridge, United Kingdom; ^2^ Consultant Endocrine Surgeon at University College of London and Great Ormond Street Hospital for Children, London, United Kingdom

## Abstract

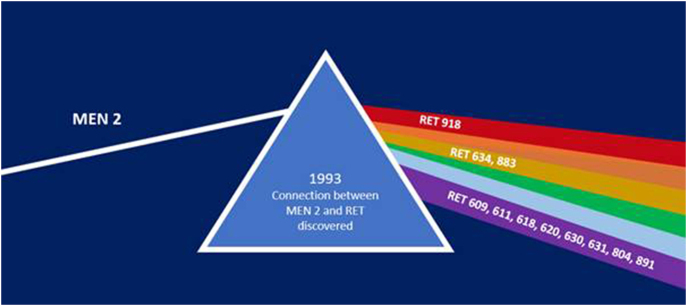

## Introduction

The 1990s were a ‘*decade mirabilis*’ when it came to the discovery of tumour suppressor genes: *NF1* in 1990, *APC* in 1991, *vHL* in 1993, *BRCA1* in 1994, *BRCA2* in 1995 and *PTEN* in 1997. In that decade, these genes were also linked to clinically characterised hereditary cancer syndromes: neurofibromatosis type 1, von Hippel–Lindau, familial polyposis coli, hereditary breast cancer and Cowden syndrome. In 1993, two articles published in *Nature* and *Human Molecular Genetics* (Donis-Keller *et al.* 1993, Mulligan *et al.* 1993) announced the connection between yet another clinically recognised syndrome, multiple endocrine neoplasia type 2 (MEN2), originally described in the 1960s (Sipple 1961), and to the *RET *(rearranged during transfection receptor tyrosine kinase) gene, previously discovered in 1985 (Takahashi *et al.* 1985). The remarkable novelty of this discovery was that, apart from linking MEN2 and RET together, it also introduced a new paradigm for tumour inheritance alternative to the ‘second hit’ Knudson hypothesis, a gain-of-function mutation causing an inherited risk of cancer (Knudson 1971).

The year 2023 marked the 30th anniversary of this discovery, and ERC celebrates this occasion with a special themed collection, appropriately named RET@Thirty. Our editorial looks back at the story of the discovery of RET as the cause of MEN2 and how this breakthrough changed the way we care for patients with this condition.

## How I got involved and getting started

### Bruce Ponder

I had no formal background in either endocrinology or clinical genetics, so it is surprising that I should set out early in my career to find the gene for MEN2. A lot of it was luck, but luck driven by instincts learnt from my time as a student at Cambridge, where – unusually for a medical student – I took a third-year option in biochemistry. It was 1964, just ten years after the double helix; my supervisor was working on the genetic code. I think I sensed then the coming revolution in biology and with it, the potential to better understand mechanisms of disease, but this lay dormant until the time arose.

Sixteen years later, helping out one day in a busy thyroid clinic led me quite by chance to MEN2. On top of the pile were two thick sets of case notes. I took them, and both were MEN2 families. Here was my opportunity. It was 1981; the human gene map was just starting; the time was right. I decided to set up a ‘Family Cancer Clinic’, starting with MEN2, to provide advice about risks and to search for the genes, and through them, gain insight into the mechanisms of disease. The project was potentially vast. At the site visit the next year, I was deemed, at best, to be mad; but someone in the Cancer Research Campaign must have supported me. (Would this be possible now, 40 years on?).

MEN2 was a good place to start. In comparison to familial breast cancer, which we moved to next, MEN2 is a well-defined, easily recognised syndrome. Many known families were already under clinical care, with effective interventions available for those at risk. This made it easier to invite them to help with research. We set up a UK Medullary Thyroid Cancer Group, including all the relevant clinical disciplines, and this expanded rapidly across Europe and beyond. Collaborations were established with other MEN2 groups, notably in the USA, with pooling of data and analysis at a series of international workshops.

The search for the MEN2 gene would depend on the human gene map. This was still to be built, using DNA probes: first, mapped to different chromosomes by physical hybridisation, and second, mapped in relation to each other on the chromosome by ‘genetic linkage’. Linkage mapping required that each ‘marker’ gene exist in two different forms, based on single base variations in DNA sequence that could be revealed by cutting of the DNA (or not) by restriction enzymes. Between each generation, at meiosis in the production of germ cells, ‘shuffling’ of genes can occur between sister chromatids. The closer the two genetic markers are on the chromosome, the less likely they are to be ‘shuffled’ apart, and the more closely they will be inherited together through generations within the family – they are said to be ‘linked’. In the search for the disease gene, ‘linkage’ of the disease with a marker places the disease gene on the map. To have greater numbers and therefore power, ideally one would score both affected and unaffected family members. But then, one had to know which apparently unaffected family members might in fact be gene carriers. An important collaborative analysis by our group was to define the age-related probability that a gene carrier would be detected by clinical examination alone, by calcitonin testing or – the most sensitive – by stimulated testing, where calcitonin is measured after intravenous injection of pentagastrin (Easton *et al.* 1989). This allowed apparently unaffected individuals to contribute to the mapping with appropriate correction for age and screening data.

By 1986, mapping had begun. The human gene map was estimated to cover 30% of the genome. A pooled analysis of data at an MEN2 workshop in Cambridge, attended by 75 participants from 14 countries, showed chromosome 10 to be the most probable location for the MEN2 gene – but only because that chromosome had, as yet, no markers, and thus, no negative data. Later that year, the Yale group reported the first, weak, positive ‘hit’: it was on chromosome 10. Progress over the next four years followed the gradual assembly of the chromosome 10 map. There was good sharing of data between groups. By early 1989, we calculated from our data 60:1 odds that the MEN2 gene lay between two markers that mapped on either side of the chromosome 10 centromere. They were still several millions of base pairs apart, and much of the DNA around the centromere consisted of large blocks of repetitive sequence, which would make more detailed mapping difficult. As the density of markers on the human gene map increased, different competitive groups often chose different markers, and comparisons and combination of data became more difficult. By mid-1992 – after seven years, now, it would take maybe a few weeks – our own linkage mapping had reached its limit. Disease mapping by linkage depends on the failure of co-inheritance of disease and marker, and the distances were now so small that such events would be rare. The odds that MEN2 lay between the markers D10S141 and D10S94 – an estimated distance of 0.5 million base pairs – were sufficient to justify building a physical map of that DNA using human DNA inserted into yeast artificial chromosomes.

But there were several putative genes in this region: how would we know which was MEN2? Here, we nearly came unstuck, until luck once more came to the rescue. The paradigm for inherited cancers at that time was the Knudson hypothesis (Knudson 1971). This sought to explain how an inherited mutation that led to cancer could be inherited without causing problems in development. Knudson postulated that the inherited mutation should be a recessive ‘loss of function’ mutation, which would be ‘covered’ by the remaining normal allele; cancers would arise only if, by chance, this second allele was also mutated in a single cell, most likely after birth (the ‘second hit’). This was shown for mutation of the *RB1* gene in retinoblastoma and mutation of *TP53* in Li–Fraumeni syndrome. In each cancer, the ‘second hit’ commonly involved a sizeable deletion in the cancer cells of the chromosome segment carrying the remaining allele. If that were the case in MEN2, we should expect to find such deletions of the MEN2 gene in a high proportion of the tumour cells, and those would be a clear signpost to the gene. But after extensive studies we had found nothing to guide us. It seemed that MEN2 might not follow this model, the inherited mutation might be dominantly acting and we might be facing the task of searching several genes within the 0.5 million base pair region to find possibly single base changes. With the technology and resources then available to us, that was a daunting task.

This is where luck again came to the rescue. Jet lagged and climbing the stairs to bed at a genetics meeting in Hilton Head, I met Mariano Barbacid, a Spanish mouse geneticist whom I knew well. ‘Hi Mariano. What’s new?’. He told me that they had just knocked out ‘a gene called ret’ in a mouse, and the mouse got something similar to Hirschsprung disease. I think he knew that ret was on human chromosome 10, but not that it was in the middle of our 0.5 million base pair interval. Nor – which I knew – that a family had been reported ten years earlier in a Canadian journal (Verdy *et al.* 1982), in which family members had both Hirschsprung and MEN2. Next day, I called the laboratory, Lois Mulligan did a heteroduplex-based mutation search in mRNA from several MEN2 families and 20/23 had RET missense mutations (Mulligan *et al.* 1993). 19 of these 20 mutations altered conserved cysteine residues lying at the boundary of the extracellular and transmembrane domains of the RET protein. Our laboratory was not expert in receptor tyrosine kinases, but others were, and they quickly pointed out the likely mechanism. The cysteines were normally engaged in intramolecular bonding to ensure the conformation of the protein, but with one mutated, the remaining one was free to cross link with the free cysteine in a neighbouring RET molecule, activating the RET tyrosine kinase. MEN2 was a dominantly acting cancer mutation.

## Surgery guided by genetics 

### Tom Kurzawinski

With a remarkable speed, by the end of the 1990s, *RET* testing became uniformly available across the United Kingdom, undoubtedly one of the many benefits of the National Health Service (NHS), which facilitated early ‘bench to bedside’ transfer of healthcare innovations. I still remember the excitement, soon after my appointment as a new Consultant Surgeon in 1997, of receiving a letter in which I was informed that two siblings referred for prophylactic thyroidectomy had MEN2 diagnosis confirmed by genetic test. Hooray, gone were the days of speculating which child inherited the condition, anxious waiting for the tumours to manifest themselves and performing unpleasant ‘stimulation tests’ when calcitonin was measured after intravenous injection of calcium or secretin. Positive genetic tests not only gave us diagnostic certainty, but we soon realised that it allowed for the prediction, with a fair degree of accuracy, of which tumours are likely to develop, and at what age. Groundbreaking publications documented amazingly strong genotype–phenotype correlations, with particular *RET* pathogenic variants leading to age-dependent penetrance of medullary thyroid cancer, phaeochromocytoma and primary hyperparathyroidism (Eng *et al.* 1996). This was truly exciting, we were able to make a diagnosis earlier, offer surgery sooner, perform less invasive operations and as a result of this, improve outcomes in patients with MEN2, especially in children.

A rear mirror view of the management of MEN2 clearly identifies the mid-1990s as the pivotal point when everything changed. Understanding MEN2 through the genetic prism of *RET* mutations was like the splitting of a light beam into rays of different colours, allowing us to see this condition not as a monolithic disease but as a cluster of different pathologies with their own trajectories, each requiring an individual approach. The management of patients with MEN2 entered a new era and became the paradigm for precision medicine. Best practice was regularly summarised by the series of international guidelines, updated according to new emerging evidence (Brandi *et al.* 2001, American Thyroid Association Guidelines Task Force *et al.* 2009, Wells *et al.* 2015). Rapidly expanding knowledge of RET biology, structure and signalling led to the development of innovative drugs designed to interrupt pathological signalling. The Times They Were A-Changing and this themed collection in ERC will feature 12 invited minireviews from international experts, celebrating that change. We hope you enjoy reading it.

## Declaration of interest

The authors declare that there is no conflict of interest that could be perceived as prejudicing the impartiality of this work.

## Funding

This work did not receive any specific grant from any funding agency in the public, commercial or not-for-profit sector.
